# Degradation of IL-4Ralpha by Immunoproteasome: implication in airway type 2 inflammation and hyperresponsiveness

**DOI:** 10.3389/fimmu.2025.1501898

**Published:** 2025-03-18

**Authors:** Niccolette Schaunaman, Diana Cervantes, Deborah A. Ferrington, Hong Wei Chu

**Affiliations:** ^1^ Department of Medicine, National Jewish Health, Denver, CO, United States; ^2^ Doheny Eye Institute, Pasadena, CA and University of California, Los Angeles, Los Angeles, CA, United States

**Keywords:** immunoproteasome, IL-4RA, type 2 inflammation, precision-cut lung slices, airway hyperresponsiveness

## Abstract

**Introduction:**

Immunoproteasome (IP) is induced by pro-inflammatory stimuli such as interferon gamma to regulate inflammation and immunity. Asthma patients with airway type 2 high inflammation (e.g., IL-13) demonstrate more eosinophils and airway hyperresponsiveness (AHR) with less interferon gamma. The role of IP in regulating airway eosinophilic inflammation and AHR has not been investigated.

**Methods:**

This study was aimed to determine how IP regulates type 2 inflammation and AHR using LMP7 (a subunit of IP) deficient mouse lungs, precision-cut lung slices (PCLS), and cultured human airway epithelial cells treated with IL-13 in the absence or presence of an IP inhibitor ONX-0914 or exogenous IP.

**Results:**

LMP7 KO mouse lungs had significantly more IL-4Rα protein expression than the wildtype (WT) mice. Following IL-13 treatment in PCLS, LMP7 KO mice had significantly more airway contraction than WT mice, which was coupled with increased eotaxin-2 levels. IP inhibition by ONX-0914 in IL-13 treated human airway epithelial cells resulted in significantly more IL-4Rα protein expression and eotaxin-3 release. IP inhibition in human PCLS significantly increased AHR.

**Conclusion:**

Collectively, these data demonstrated that IP promotes degradation of IL-4Rα, while inhibits type 2 inflammation and AHR. Enhancement of IP expression or activity may serve as an alternative approach to reduce the severity of type 2 inflammation and AHR.

## 1 Introduction

Asthma is a chronic respiratory disease affecting approximately 25 million people, or 7.7% of all Americans (1). Of those suffering from asthma, allergic asthma is the most prevalent phenotype (2, 3). Allergic asthma is associated with wheezing, bronchoconstriction, and type 2 or allergic inflammation characterized by the presence of type 2 cytokines, such as IL-13 and IL-4 (4, 5), which induce the production of chemokines involved in the recruitment and activation of inflammatory cells such as eosinophils (6–9). Increased levels of eosinophils have been associated with increased asthma morbidity such as worsened asthma exacerbations and airways hyperresponsiveness (10–15).

The immunoproteasome (IP) is proteolytic machinery derived from the constitutive proteasome (16, 17). In the presence of pro-inflammatory stimuli such as interferon gamma (IFN-γ), IP subunits LMP2, MECL-1, and LMP7 are induced to replace β1, β2, and β5 subunits in the constitutive proteasome (16). Interestingly, in type 2 inflammation-high asthmatics, IFN-γ levels are low compared to type-2 low asthmatics (18–21), suggesting a potential deficiency of IP. IP is known for its ability to cleave peptides for antigen presentation on major histocompatibility complex (MHC) class I molecules in T cells (22, 23). In addition, IP has been shown to regulate inflammatory responses to viral infection and NF-kB signaling (22–24). The role of IP in regulating allergic inflammation remains unknown. We have recently found that IP deficient mice challenged with house dust mite and then infected with rhinovirus showed significantly more eosinophilic inflammation than their wild-type counterparts (25). The mechanism of how IP inhibits type 2 inflammation was not determined. Chen et. al., demonstrated that LMP7 deficiency enhances M2 macrophage polarization through the regulation of the IL-4 signaling pathway (26). In this study, they showed that LMP7 deficient alveolar macrophages increased STAT6 activation and IRF4 expression, which was associated with an increase in IL-4Rα, a receptor shared by both IL-4 and IL-13 (27–30). However, the authors did not address how IP regulated IL-4Rα expression.

In the present study, we hypothesize that IP reduces lung IL-4Rα levels, leading to reduction in type 2 inflammation, a key contributor to airway hyperresponsiveness. To test this hypothesis, we utilized LMP7 deficient mouse lungs or human donor lungs to generate precision-cut lung slices (PCLS), which allows us to maintain the three-dimensional structure of the lung and measure airway contraction induced by stimuli such as IL-13. Additionally, we performed cell culture experiments to examine the role of IP in human airway epithelial IL-4Rα expression and eosinophils chemokine production with or without IL-13 treatment.

## 2 Materials and methods

### 2.1 Mice

Wild-type (WT) C57BL/6 mice were purchased from the Jackson Laboratories. LMP7 knockout (KO) mice on a C57BL/6 background were kindly provided by Dr. Deborah A. Ferrington at the Doheny Eye Institute, CA. All mice were bred at the National Jewish Health (NJH) biological resource center (BRC) under pathogen-free housing condition. All animal studies were reviewed and approved by the Institutional Animal Care and use Committee at NJH.

### 2.2 Mouse model of house dust mite challenge

To induce mouse allergic airway inflammation, WT and LMP7 KO mice (8 – 12 weeks of age, gender matched) were intranasally sensitized with 10µg/mouse of HDM (Greer laboratories, Lenoir, NC) or 50µl of phosphate-buffered saline (PBS) on day 0 and 7. Mice were then challenged once a day for three consecutive days (day 14-16) with 10µg of HDM or 50µl PBS (25, 31, 32)). 72 hours after the last HDM challenged mice were sacrificed. The left lung was used for IL-4Rα protein quantification via western blot as well as for cytokine mRNA expression. One lobe of the right lung was fixed in 10% formalin for immunofluorescent staining.

### 2.3 Preparation of precision-cut lung slices

#### 2.3.1 Mouse PCLS

Naïve WT and LMP7 KO mice were euthanized by intraperitoneal injection of pentobarbital sodium (Fatal-Plus) in sodium chloride. Lungs were inflated with 1.5% low-melting agarose and sliced into consecutive 250µm thickness sections using a Compresstome^®^ VF-300 vibratome (Precisionary Instruments, Natick, MA).

#### 2.3.2 Human PCLS

The upper lobes of the right lung from eight healthy, non-smoking donors were obtained from the International Institute for the Advancement of Medicine (IIAM, Philadelphia, PA), and Donor Alliance of Colorado (Denver, CO). All donor lungs were selected based on non-smoking status and no history of lung disease/infection. The detailed donor demographic information is given in **Table 1**. Lungs were inflated as previously published (33) and sliced into consecutive 300µm thickness sections. The Institutional Review Board (IRB) at NJH approved our studies as meeting requirements of exempt human subject research.

**Table 1:** Demographic information of human donors for the PCLS experiment.

Subject Number	Age (years)	Sex	Smoking History	Cause of death
1	60	Male	Nonsmoker	Stroke
2	32	Female	Nonsmoker	Overdose
3	27	Female	Nonsmoker	Head injury
4	56	Female	Nonsmoker	Brain aneurysm
5	20	Male	Nonsmoker	Stroke
6	62	Female	Nonsmoker	Head injury
7	65	Male	Nonsmoker	Head injury
8	58	Male	Nonsmoker	Overdose

#### 2.3.3 Culturing of PCLS

Both human and mouse slices were transferred to 24-well plates containing Dulbecco’s Modified Eagle’s Medium (DMEM, ThermoFisher Scientific, Waltham, MA) with antifungal agents and antibiotics and incubated in a humidified incubator at 37°C supplemented with 5% CO_2_.

### 2.4 Treatment of PCLS

#### 2.4.1 Mouse PCLS

24 hours after slicing, mouse PCLS were washed with 1X PBS (Fisher Scientific, Hampton, NH), and then treated with 25ng/ml of recombinant murine IL-13 (PeproTech, Cranbury, NJ). Supernatant was harvested 72 hours after IL-13 treatment, and slices underwent methacholine challenge for airway hyperresponsiveness measurement.

#### 2.4.2 Human PCLS

24 hours after slicing, human PCLS were washed with 1X PBS (Fisher Scientific, Hampton, NH), and then pre-treated with 100nM ONX-0914, an immunoproteasome inhibitor (APExBio, Houston, TX), for two hours followed by treatment with 25ng/ml of recombinant human IL-13. Supernatant was harvested 72 hours after IL-13 treatment, and slices underwent methacholine challenge for airway hyperresponsiveness measurement.

### 2.5 Airway hyperresponsiveness measurement

AHR measurement was performed as we previously published (34). After removing the supernatant, 500µl of warmed DMEM with antifungal agents and antibiotics was added back to PCLS. For both mouse PCLS and human PCLS, baseline images of the airways were taken, followed by adding increasing doses of methacholine (McH) for a final concentration of 1, 10, 100, and 1,000µM to each well. After 30 seconds of each dose of McH, images of airways were taken. The area (relative pixel number) within the lumen was traced using the image J freehand tool, and the percent airway constriction was calculated as (1 – post McH area/baseline area) x 100. The number of airways measured for AHR was between 3-6 per condition for each donor/mouse.

### 2.6 Culture of human tracheobronchial epithelial cells

HTBE cells were processed as previously described (35) from deidentified donor lungs that were not suitable for transplantation under a protocol approved by the IRB at NJH. The selected donor lungs were obtained through the IIAM and Donor Alliance of Colorado, and all were non-smokers. The HTBE cells were isolated by enzymatic digestion from the distal region of the trachea and proximal regions of the main bronchi. Freshly isolated HTBE cells were cultured up to one week on 100mm dishes in BronchiaLife culture medium (Lifeline Cell Technology, Frederick, MD). HTBE cells were trypsinized and frozen in liquid nitrogen for future use in BronchiaLife medium supplemented with 30% Fetal Bovine Serum (FBS, SeraPrime, Fort Collins, CO), and 10% dimethyl sulfoxide.

### 2.7 Treatment of HTBE lysate with exogenous IP

HTBE cells from seven healthy donors were expanded on 100mm culture dishes in BronchiaLife media. Once 100% confluent, cells were trypsinized and lysed in 100µl of radioimmunoprecipitation assay buffer (RIPA). 50µg of lysed protein was incubated with either 0nM, 5nM, or 20nM of whole IP isolated from human spleens (South Bay Bio, San Jose, CA) in 100mM BTP (pH 7.5), 100mM KCL, 10mM EGTA, and 0.035% sodium dodecyl sulfate (SDS) for 5 hours at 37°C. After 5 hours of IP treatment, the mixture of cell lysate and IP was frozen immediately at -80°C for western blotting later.

### 2.8 Treatment of HTBE cells with an IP inhibitor and IL-13

HTBE cells from six healthy donors were expanded and cultured on 24-well plates in BronchiaLife media. Once 80% confluent, cells were pre-treated with 100nM ONX-0914 for two hours. After two hours, cells were treated with 2.5µg/ml of an anti-IL4Rα antibody (R&D Systems, Minneapolis, MN) or IgG control for 30 minutes. After 30 minutes, cells were treated with 10ng/ml of recombinant human IL-13 (PeptroTech, Cranbury, NJ). Cells and supernatants were harvested both 5 hours after ONX-0914 treatment and 72 hours after IL-13 treatment. 5 hours was chosen to match the incubation time of exogenous IP with airway epithelial cell lysates done above.

The doses of ONX-0914 and IL-13 were selected based on our previous publications (36–39). We chose the dose of the anti-IL4Rα antibody from our dose-response optimization study that showed the maximal reduction of eotaxin-3 levels at 2.5µg/ml in IL-13-stimulated human airway epithelial cells (data not shown).

### 2.9 Western blotting

Cells were lysed in RIPA buffer with Halt protease and phosphatase inhibitor cocktail 100x (ThermoFisher, Waltham, MA). Equal amounts of proteins were loaded and separated by SDS-PAGE, transferred onto PVDF membranes, blocked with blocking buffer, and incubated with the following primary antibodies overnight at 4°C: IL4R Polyclonal antibody (Anti-human, Proteintech Group, Inc., Rosemont, IL), LMP2 (Abcam, Cambridge, United Kingdom), LMP7 (Proteintech Group Inc. Rosemont, IL), IL-4Rα Antibody (Anti-mouse, Santa Cruz Biotechnology, Dallas, TX), and Beta-Actin antibody (Santa Cruz Biotechnology, Dallas, TX). After washes in PBS with 0.1% Tween-20, membranes were incubated with the appropriate horseradish peroxidase (HRP)-linked secondary antibodies and developed using a Fotodyne imaging system (Fotodyne Inc., Harland, WI).

### 2.10 Immunofluorescent staining of IL-4R in the lung

Immunofluorescent (IF) staining was performed on formalin-fixed and paraffin-embedded lung tissue sections from WT and LMP7 KO mice treated with either PBS or challenged with HDM. Sections cut at 5µm were deparaffinized and immersed in pre-heated (95°C) 10mM sodium citrate buffer with 0.05% Tween 20 (pH 6.0) for 20 minutes for antigen retrieval. Slides were incubated with an anti-IL4Rα antibody (ThermoFisher Scientific, Waltham, MA) at 1:100 dilution in 0.1% Triton X-100 in PBS overnight at 4°C, and then incubated with a secondary anti-rabbit antibody conjugated with AF594 at a 1:500 dilution at room temperature for one hour. Slides were then mounted with anti-fade mounting solution with 4’,6-diamidino-2-phenylindole (DAPI) (ThermoFisher). IF staining was imaged using the Revolve microscope (Echo, San Diego, CA). Images were taken at 200x using the same overlay settings for each tissue section.

### 2.11 Reverse transcription and quantitiative real-time PCR

RNA was extracted from homogenized lung tissue as decribed previously (25, 31, 39, 40). Briefly, RNA was isolated using the TRIzol reagent method, and then reversely transcribed to cDNA.

Taqman Gene Expression Assay (ThermoFisher, Waltham, MA) was used to determine mRNA relative levels of IL-13 and IL-4. Target gene expression was normalized to the housekeeping gene 18S rRNA. The comparative threshold cycle method (ΔΔCt) was applied to determine the relative levels of target genes.

### 2.12 Statistical analysis

GraphPad PRISM version 10.0 software was used for all statistical analysis. Nonparametric data were analyzed using the Kruskal-Wallis test for multiple comparisons, and the Mann-Whitney test for two-group comparisons. A p-value <0.05 was considered statistically significant.

## 3 Results

### 3.1 Immunoproteasome deficiency increases IL-4Rα protein levels in mouse lungs

**Figure 1:**
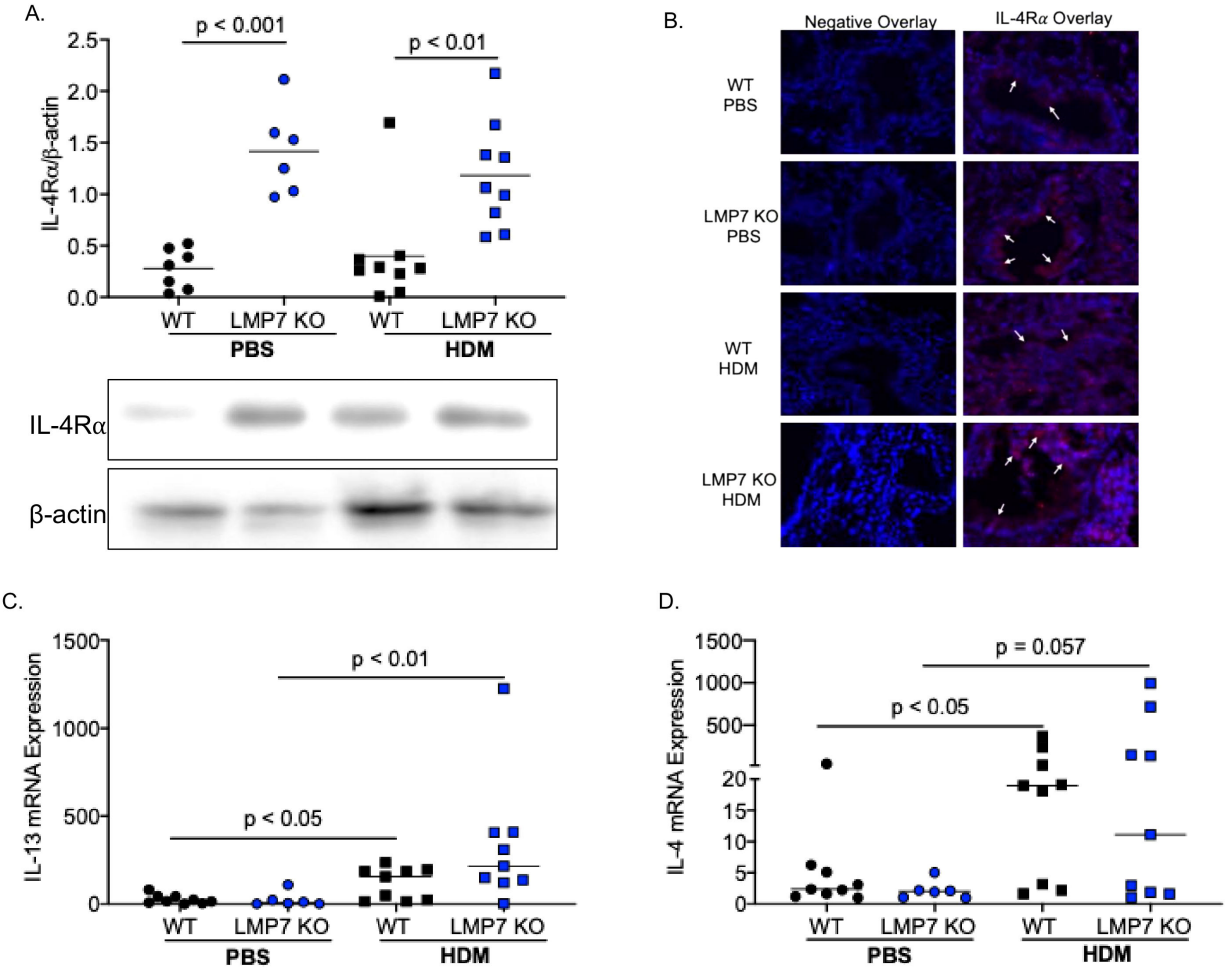
LMP7 deficient mice have significantly more IL-4Rα in homogenized lung tissue. **(A)** LMP7 KO mice treated with PBS or house dust mite (HDM) had significantly more IL-4Rα protein present in the lung tissue compred to WT mice. **(B)** IL-4Rα immunofluorescent staining of WT and LMP7 KO lung tissue treated with or without HDM; white arrows represent IL-4Rα staining (red color) on airway epithelial cells. HDM challenge significantly increased IL-13 **(C)** and IL-4 **(D)** mRNA levels in both WT and LMP7 KO mice, however there was not statistical difference between WT and LMP7 KO.

We have previously shown that allergen challenged IP deficient mice had significantly more eosinophils and eotaxin-2 compared to WT mice (25). To determine the possible mechanisms by which IP regulates type 2 inflammation, we measured IL-4Rα in lungs of WT and LMP7 KO mice treated with either PBS or house dust mite (HDM). HDM has not been found to regulate IL-4Rα levels (41). Similarly in our model, HDM challenge did not increase IL-4Rα levels in either strain of mice. However, LMP7 KO mice had significantly more IL-4Rα protein expression in homogenized lung tissue compared to WT mice (**Figure 1A**), both at baseline as well as after HDM challenge. This data supports our previous findings that LMP7 KO mice are more susceptible to type 2 inflammatory responses (e.g., eosinophil recruitment) during allergen challenges. To localize IL-4Rα in the lung, immunofluorescent staining was performed. IL-4Rα signal (**Figure 1B**) was observed in airway epithelium as well as in the alveolar areas which may include immune cells and structural cells. LMP7 KO mice treated with either PBS or HDM seem to have more positive IL-4Rα staining in airway epithelial cells.

To determine if the increased lung eosinophil recruitment in LMP7 KO mice may be induced by heightened type 2 cytokine production, we measured mRNA expression of IL-13 and IL-4. As expected, HDM challenge significantly increased IL-13 levels in WT and LMP7 KO mice (**Figure 1C**) (42, 43). However, there was no significant difference in IL-13 mRNA levels between WT and LMP7 KO mice. A similar trend was seen with IL-4 mRNA expression (**Figure 1D**). Our data suggests that excessive eosinophilic inflammation seen in LMP7 KO mice may be attributed to the up-regulation of IL-4Rα and subsequent type 2 inflammatory response.

### 3.2 IP deficiency enhances airway hyperresponsiveness in mouse precision-cut lung slices

**Figure 2:**
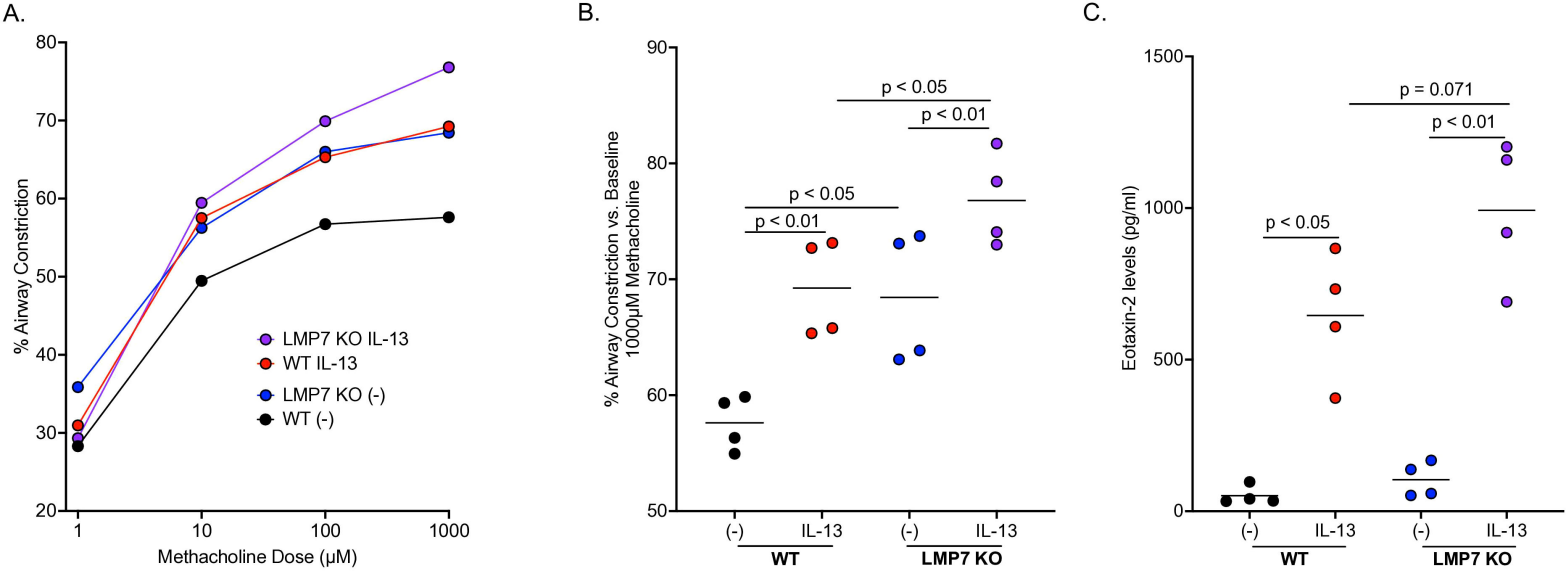
LMP7 deficiency enhances airway hyperresponsiveness in mouse precision-cut lung slices (PCLS) exposed to IL-13. WT and LMP7 KO mouse lungs were inflated, sliced at 250µm, and treated with or without 25ng/ml of IL-13 for 72 hours. Airway contraction with increasing doses of methacholine was measured with each symbol representing the average airway contraction of all experiments **(A)**. LMP7 KO mice had significantly more airway contraction compared to WT mice, which was further increased by IL-13 **(B)**. Eotaxin-2 levels were increased in PCLS by IL-13, with LMP7 KO vs. WT lung tissue having significantly higher eotaxin-2 levels **(C)**. The symbols represent individual experiment with 3 – 8 technical replicates per experiment.

To determine if IP deficiency enhances AHR, a common feature associated with type 2 inflammation, WT and LMP7 KO mouse PCLS were treated with IL-13 for 72 hours after which airway lumen area was measured following methacholine challenges. **Figure 2A** shows a dose-dependent increase in airway contraction with each increasing dose of methacholine. As previously reported (44–48), IL-13 treatment in WT mouse PCLS significantly increased AHR compared to control treated slices (**Figure 2B**). LMP7 KO PCLS had significantly more airway contraction compared to WT slices at baseline, which was further enhanced after IL-13 treatment as AHR in IL-13 treated LMP7 KO PCLS was significantly greater than WT PCLS. Eotaxin-2, an eosinophil chemokine, was upregulated by IL-13 in WT and LMP7 KO PCLS compared to controls (**Figure 2C**). IL-13 treated LMP7 KO PCLS also had more eotaxin-2 compared to IL-13 treated WT slices.

### 3.3 IP inhibition increases IL-4Rα and eotaxin-3 in IL-13-stimulated human airway epithelial cells

**Figure 3:**
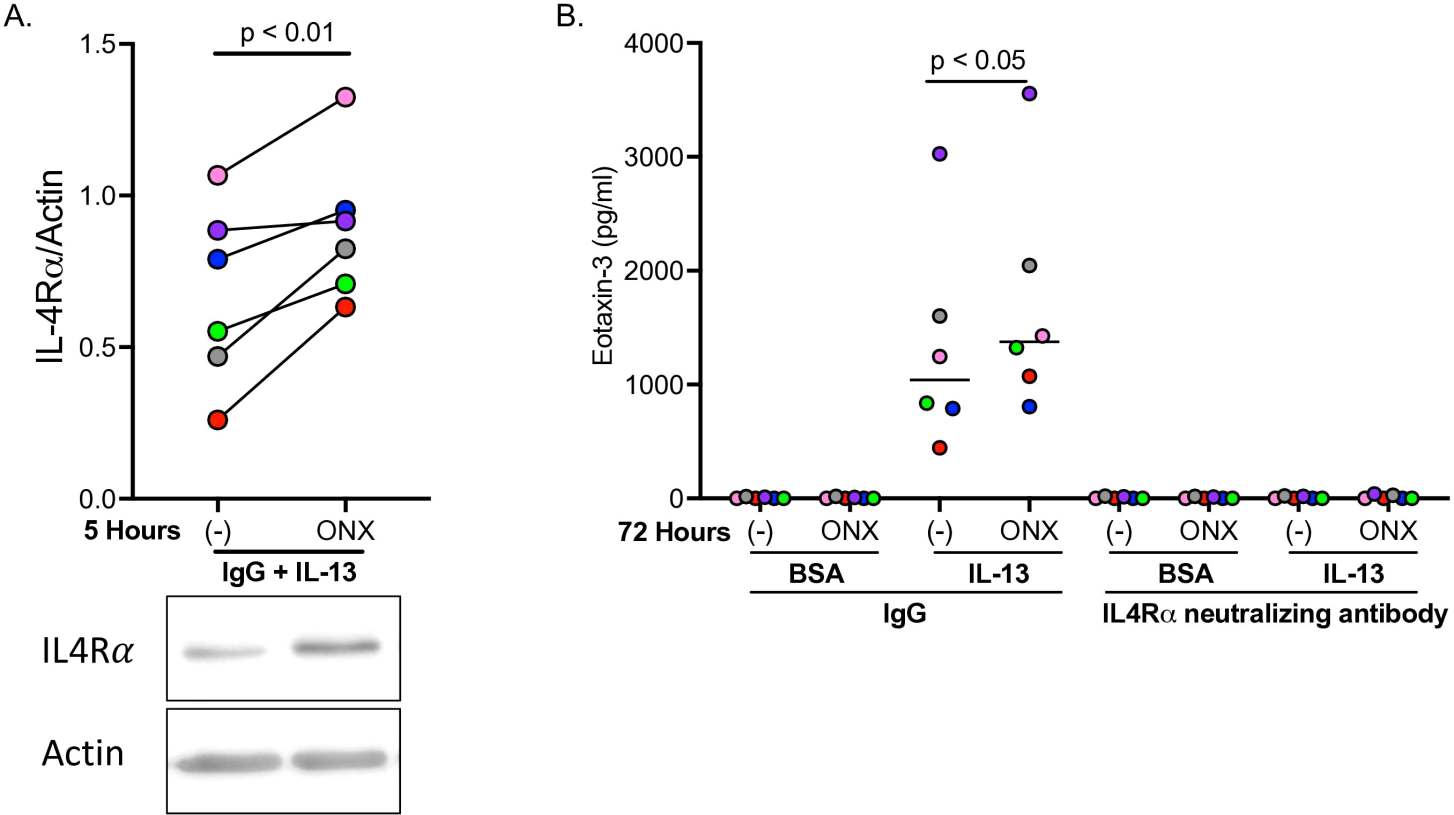
Inhibition of immunoproteasome (IP) increases IL-4Rα and eotaxin-3 in IL-13-stimulated human airway epithelial cells. Tracheobronchial epithelial cells from n=6 healthy donors were pre-treated with immunoproteasome LMP7 inhibitor ONX-0914, then given anti-IL4Rα antibody followed by IL-13 treatment. IP inhibition in IL-13 treated cells significantly upregulated IL-4Rα protein levels five hours after ONX-0914 treatment **(A)**, as well as significantly increased eotaxin-3 levels after 72 hours **(B)**. Anti-IL4Rα antibody significantly inhibited IL-13-induced eotaxin-3.

To further establish a role for IP during type 2 inflammation in human airway epithelial cells, IL-4Rα protein levels were measured five hours after HTBE cells were exposed to IL-13 with and without ONX-0914. ONX-0914 in IL-13 treated cells significantly increased IL-4Rα levels compared to IL-13 treatment alone (**Figure 3A**). Eotaxin-3, and eosinophil chemokine upregulated by IL-13, was increased by IL-13 at 72 hours (**Figure 3B**). ONX-0914 in IL-13-stimulated cells significantly enhanced eotaxin-3 production. Importantly, when cells were treated with an anti-IL4Rα antibody in the presence of ONX-0914 and IL-13, eotaxin-3 levels were completely inhibited. Our data suggests that IP inhibition increases IL-4Rα protein expression, which is critical to eotaxin production.

### 3.4 IP directly reduces IL-4Rα levels in cultured human airway epithelial cells

**Figure 4:**
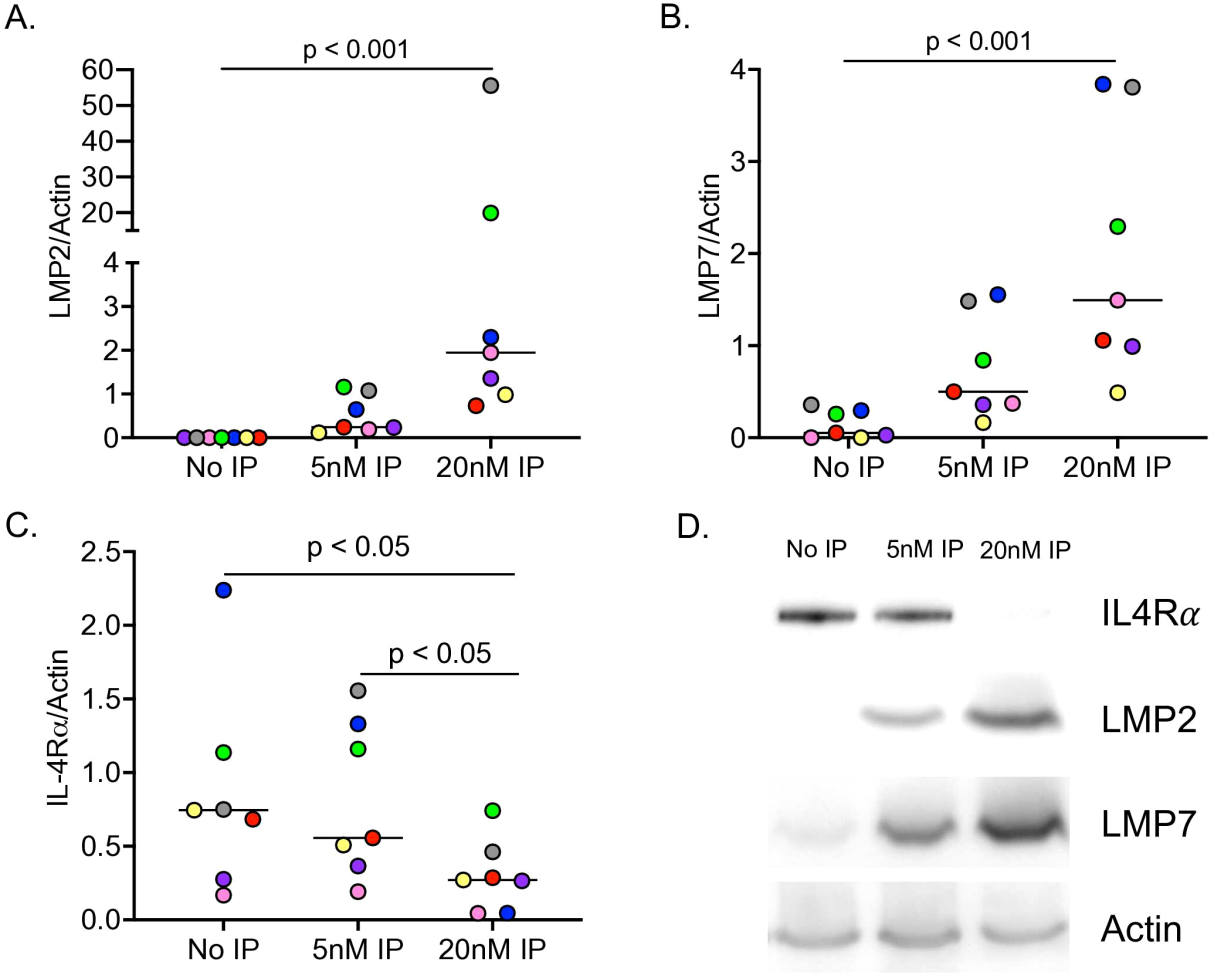
The immunoproteasome (IP) degrades IL-4Rα in human airway epithelial cell lysates. Tracheobronchial epithelial cells from n=7 healthy donors were incubated with 5nM and 20nM of exogenous IP for 5 hours at 37°C. IP it increased LMP2 **(A)** and LMP7 **(B)** protein expression, while it decreased IL-4Rα protein expression in a dose-dependent manner **(C)**. Representative western image of IP degrading IL-4Rα and increasing IP subunits **(D)**.

To determine the direct effect of IP on IL-4Rα protein content, IP isolated from human spleen was incubated with naïve HTBE cells from seven healthy donors for five hours at 37°C. Accordingly, LMP2 and LMP7 (subunits of IP) protein levels increased in a dose-dependent manner following incubation with exogenous IP at 5nM and 20nM (**Figures 4A, B**). While 5nM IP trended to decrease IL-4Rα protein levels, 20nM IP significantly decreased IL-4Rα levels compared to control and the 5nM IP concentration (**Figure 4C**). Representative western blot images for IL-4Rα, LMP2, and LMP7 protein expression can be seen in **Figure 4D**.

### 3.5 IP inhibition increases AHR in human PCLS

**Figure 5:**
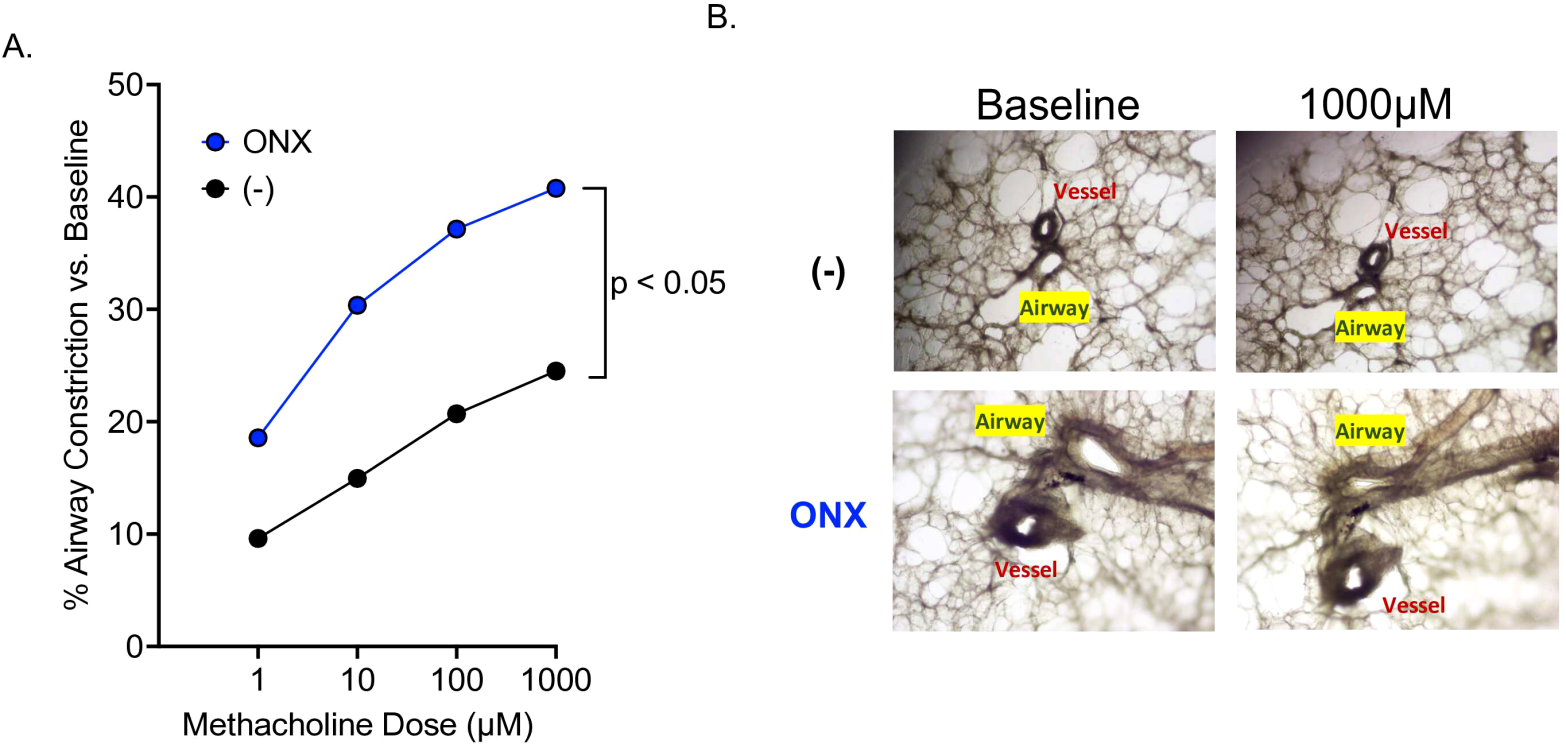
IP inhibition in human PCLS significantly increases airway hyperresponsiveness. Lung from n=8 healthy human lung donors were inflated with low melting agarose, cored, sliced to 300µM, and treated with or without ONX-0914. **(A)** Airway contraction data. **(B)** Representative images of airway contraction in PCLS.

As we observed the enhanced AHR in IP deficient mouse PCLS even without IL-13 treatment, we tested if IP deficiency in human distal lungs increases AHR. PCLS from eight healthy donors were treated with or without ONX-0914 for 72 hours before measuring AHR. IP inhibition by ONX-0914 significantly increased airway contraction as compared to control slices (**Figures 5A, B**).

## 4 Discussion

This is the first report studying the mechanism of regulation of type 2 inflammation by IP, and the impact of IP deficiency on airway hyperresponsiveness. Our results suggest that degradation of IL-4Rα and reduced IL-13-induced eosinophil chemokine production. In addition, IP deficiency, specifically LMP7 deficiency, contributed to airway contraction in the absence or presence of IL-13 stimulation.

The role of IP in allergic or type 2 inflammation remains controversial. Several research groups have reported differing roles of IP on eosinophilic inflammation depending on types of allergens given. Oliveri et. al., found that mice challenged with ovalbumin and an IP inhibitor had less eosinophils present than vehicle control mice. However, when challenged with house dust mite (HDM) and an IP inhibitor, eosinophil levels were not different from vehicle control mice (49). Another group found similar results when using LMP7 deficient mice and a high dose of HDM (50). Lack of mechanistic studies into how IP directly regulates allergic, or type 2 inflammation may in part explain the above controversial or inconsistent findings. In this study, we clearly demonstrated that IP regulates IL-4Rα protein expression in human airway epithelial cells as IP inhibitor treatment in IL-13-stimulated human airway epithelial cells increased IL-4Rα and eotaxin-3 levels. Moreover, this was evident in our LMP7 deficient mouse model where IL-4Rα levels were increased in LMP7 deficient lung tissue. This increase was not coupled with an increase in type 2 cytokines IL-13 or IL-4 in mice, suggesting that IP does not appear to up-regulate the expression or production of typical type 2 cytokines (e.g., IL-4 and IL-13). Instead, IP may inhibit the signaling of type 2 cytokines through IL-4Rα, such as eotaxin-2 production as we previously reported. Eotaxin-2 production is highly dependent on IL-4/IL-13 and IL-4Rα signaling and is critical to lung eosinophil recruitment and inflammation (51, 52).

Many biologics have been developed to combat type 2 inflammation and allergic asthma, with IL-4Rα being one of the main targets, since both IL-13 and IL-4 utilize it as a receptor (27–30). Our data shows that IP inhibition in human epithelial cells treated with an anti-IL-4Rα neutralizing antibody abolished IL-13-induced eotaxin-3 levels. While little is known about IL-4Rα regulation by IP or proteasome, Wei et. al., found that ubiquitination-mediated proteasomal degradation of IL-4Rα is important in controlling airway inflammation (53). The IP is proteolytic machinery that has been classically considered to play a role in antigen presentation (22, 23) by cleaving different peptides to initiate adaptive immune responses (24, 54). However, multiple studies support a more general role for IP in responding to stress (55–59). Here, we have improved our understanding of how IP may regulate type 2 inflammation by showing the inhibitory role of IP in regulating IL-4Rα content. Increased IL-4Rα expression in airway epithelium has been reported in asthma, particularly atopic/allergic asthma (60). Although genetics and other factors may contribute to increased IL-4Rα in asthma, investigating the role of IP is critical for enhancing our understanding of this important mechanism. Given that IFN-γ induces IP and IFN-γ levels are low in type 2 inflammation-high asthmatics (18–21), it is possible that IP deficiency may exist in type 2 inflammation-high asthmatics. This speculation needs to be confirmed in future work, but reduced IP may offer a new mechanism for type 2 inflammation. Severe chronic obstructive pulmonary disease (COPD) has been associated with a decrease in IP levels (61, 62). Interestingly, impaired IFN signaling has been reported in COPD patients with viral infection (63). Thus, it appears that IFN supplementation may be appropriate in asthma or COPD patients with IP deficiency to attenuate type 2 inflammation.

Airway obstruction is a salient feature of asthma, but the role of IP in airway obstruction or AHR has not been tested. By leveraging the PCLS model where the three-dimensional structure of the lung is maintained, we found that LMP7 deficiency enhanced AHR, in the absence or especially in the presence of IL-13. Even without IL-13 stimulation or at the baseline, LMP7 KO mouse PCLS had significantly more airway contraction than WT slices. This was mirrored in our human PCLS model where IP inhibition by ONX-0914 resulted in significantly more airway contraction. How IP contributes to AHR remains to be determined. One explanation could be related to IL-4Rα-mediated signaling such as smooth muscle contractility and mucus production in the presence of IL-13 stimulation. We still do not know why IP deficiency alone (no IL-13 stimulation) increases AHR. Whether IP deficiency in the lung increased the activity (e.g., release of acetylcholine) of parasympathetic nerves and smooth muscle contraction warrants further investigation. Calcium signaling is critical to airway smooth muscle contraction (64). Proteasome inhibitor may contribute to disruption of intracellular calcium homeostasis (65). Whether IP deficiency at the baseline affects calcium signaling and subsequently enhance AHR at the baseline may need to be tested.

One limitation of our study is that while IP reduces airway epithelial IL-4Rα content, the exact mechanisms (direct vs. indirect) were not determined. Further studies could investigate the direct mechanisms by determining if immunoproteasome components (e.g., LMP7) interact with IL-4Ra and ubiquitinate it, and target IL-4R1 for ubiquitination-mediated proteasomal degradation. Regarding indirect mechanisms, we could test if IP deficiency decreases the expression of other mediators involved in IL-4Rα degradation, including STIP1 homology and U-Box containing protein 1 (STUB1), a chaperone-dependent E3 ubiquitin ligase. In a previous study, STUB1 was shown to promote IL-4Rα degradation (53). Additionally, our study only examined IP-mediated IL-4Rα under IL-13. Adding IL-4, another type 2 cytokine that shares IL-4Rα as a receptor, would further our understanding of IP in the regulation of type 2 inflammatory responses. Another limitation is that we did not use a mouse asthma model to determine the role of IP in AHR. Nonetheless, the PCLS model allowed us to accurately measure airway contraction. Finally, future studies are needed to determine if IP expression and/or activity exist in asthmatics with type 2 inflammation, but not with type 1 inflammation.

By utilizing IP deficient mouse lungs and PCLS models, we have demonstrated that immunoproteasome is a negative regulator of IL-4Rα expression and type 2 inflammation, as well as airway hyperresponsiveness. Enhancement of IP expression or function may be therapeutically beneficial to reduce the severity of asthma in a subset of patients who present with high levels of type 2 inflammation in the airways.

## Data Availability

The original contributions presented in the study are included in the article/supplementary material. Further inquiries can be directed to the corresponding author.
